# Vibration Sensitivity Reduction of Micromachined Tuning Fork Gyroscopes through Stiffness Match Method with Negative Electrostatic Spring Effect

**DOI:** 10.3390/s16071146

**Published:** 2016-07-22

**Authors:** Yanwei Guan, Shiqiao Gao, Haipeng Liu, Lei Jin, Yaping Zhang

**Affiliations:** 1 State Key Laboratory of Explosion Science and Technology, Beijing Institute of Technology, Beijing 100081, China; guanyanwei2006@163.com (Y.G.); gaoshq@bit.edu.cn (S.G.); yapingzhang1@163.com (Y.Z.); 2 School of Mechatronical Engineering, Beijing Institute of Technology, Beijing 100081, China; jinlei@bit.edu.cn

**Keywords:** vibration sensitivity, micromachined tuning fork gyroscopes, coordinate transformation method, stiffness match method, negative electrostatic spring effect

## Abstract

In this paper, a stiffness match method is proposed to reduce the vibration sensitivity of micromachined tuning fork gyroscopes. Taking advantage of the coordinate transformation method, a theoretical model is established to analyze the anti-phase vibration output caused by the stiffness mismatch due to the fabrication imperfections. The analytical solutions demonstrate that the stiffness mismatch is proportional to the output induced by the external linear vibration from the sense direction in the anti-phase mode frequency. In order to verify the proposed stiffness match method, a tuning fork gyroscope (TFG) with the stiffness match electrodes is designed and implemented using the micromachining technology and the experimental study is carried out. The experimental tests illustrate that the vibration output can be reduced by 73.8% through the stiffness match method than the structure without the stiffness match. Therefore, the proposed stiffness match method is experimentally validated to be applicable to vibration sensitivity reduction in the Micro-Electro-Mechanical-Systems (MEMS) tuning fork gyroscopes without sacrificing the scale factor.

## 1. Introduction

MEMS vibrational gyroscopes are a kind of inertial sensor for measuring angular rate or angle, based on an energy transfer of two vibrational modes with the Coriolis effects [1]. Micromachined gyroscopes are widely used in the military and civilian areas, because of their small size, low cost, high precision, low power consumption, batch production and easy integration. The performance characteristic of MEMS gyroscopes such as the sensitivity, resolution, bias, and bandwidth are significantly improved owing to a high quality factor which ranges from several hundred in air to hundreds of thousands in a vacuum [2,3,4,5,6,7]. The high Q-factor not only improves the gyro performance but also amplifies the vibration amplitudes at some frequencies. Thus, the external vibration has a big effect on the gyroscope’s reliability and robustness [8,9]. 

A MEMS tuning fork gyroscope is a very common type to cancel the external linear vibration output using two identical masses that vibrate in anti-phase [10,11,12,13,14]. The linear vibration is a common-mode one caused by the external environmental vibration. Since imperfect fabrication induces structural mismatch, the output errors will be caused by the linear vibration, which is referred as “vibration sensitivity” or “vibration output”. To reduce the vibration output induced by the process imperfection, a large frequency separation between the in- and anti-phase modes needs to be implemented by increasing the coupling stiffness, but this approach reduces the sensitivity of TFGs [15,16]. In order to not sacrifice the sensitivity and to reject the vibration output, increasing the in-phase mode frequency above the anti-phase and improving the frequency separation are necessary via different coupling methods between two tines [17,18,19]. All of the above methods take advantage of the structural designs to reject the vibration output. However, how to reduce the vibration output using a control circuitry is still not studied. The negative electrostatic spring effect was used to guarantee the stiffness match of two tines in the other work, which was focused on the electrostatic regulation of quality factor [20]. The purpose of this paper is different and is focused on how to estimate the vibration output caused by the external linear vibration with the negative electrostatic spring effect. 

This paper proposes a stiffness match method to reduce the vibration sensitivity using the stiffness match electrodes. A theoretical model of the stiffness match method is established and the vibration output of the non-ideal TFG is analyzed in Section 2. Section 3 gives the detailed design and fabrication of tuning fork gyroscope as well as the stiffness match electrodes. Experimental study on the vibration output under the different stiffness mismatch is carried out and the comparisons with experimental and theoretical solutions are shown in Section 4. In Section 5, the discussion is given. Section 6 gives the conclusions.

## 2. Theoretical Analysis of Stiffness Match Method

**Figure 1:**
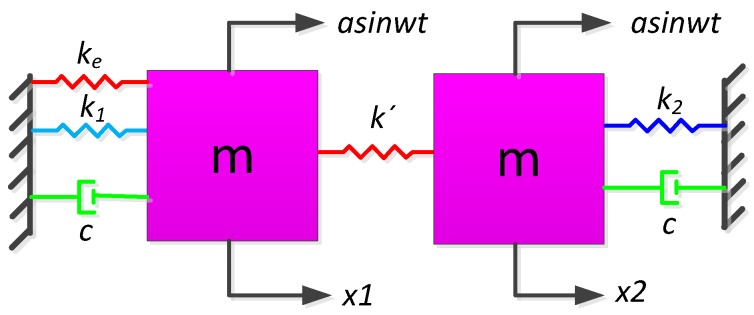
The model of the non-ideal TFG with the match stiffness.

The non-ideal TFG model with the match stiffness is shown in Figure 1. The dynamic is governed by the following.

Left tine:

(1) $$m{\ddot{x}}_{1}+c{\dot{x}}_{1}+\left(k_{1}+k_{e}\right)x_{1}+k^{\prime }(x_{1}-x_{2})=ma\sin wt$$

Right tine:

(2) $$m{\ddot{x}}_{2}+c{\dot{x}}_{2}+k_{2}x_{2}+k^{\prime }(x_{2}-x_{1})=ma\sin wt$$

Subtracting Equation (2) from Equation (1):

(3) $$m{\ddot{x}}_{1}+c{\dot{x}}_{1}+\left(k_{1}+k_{e}\right)x_{1}+k^{\prime }(x_{1}-x_{2})-m{\ddot{x}}_{2}-c{\dot{x}}_{2}-k_{2}x_{2}-k^{\prime }(x_{2}-x_{1})=0$$

Adding Equations (1) and (2):

(4) $$m{\ddot{x}}_{1}+c{\dot{x}}_{1}+\left(k_{1}+k_{e}\right)x_{1}+m{\ddot{x}}_{2}+c{\dot{x}}_{2}+k_{2}x_{2}=2ma\sin wt$$

where m and c are the mass and damping of each tine, respectively; $k_{1}$ and $k_{2}$ denote the springs stiffness and $k^{\prime }$ is the coupling stiffness between the two tines; $k_{e}$ denotes the match stiffness; $x_{1}$ and $x_{2}$ are the displacement; and asinwt is the external common mode acceleration, in which a denotes the amplitude and w is the angular frequency.

To obtain the anti-phase vibration output, a coordinate transformation is used as follows:

(5) $$x_{an}=x_{1}-x_{2},\;x_{in}=x_{1}+x_{2}$$

Substituting Equation (5) into Equations (3) and (4):

(6) $${\ddot{x}}_{an}+\frac{w_{an}}{Q_{an}}{\dot{x}}_{an}+w_{an}^{2}x_{an}=-\frac{\Delta k+k_{e}}{2m}x_{in}$$

where $w_{an}=\sqrt{\frac{k_{an}}{2m}}$, $k_{an}=k_{1}+k_{2}+k_{e}+4k^{\prime }$, $w_{in}=\sqrt{\frac{k_{in}}{2m}}$, $k_{in}=k_{1}+k_{2}+k_{e}$, $Q_{an}=\frac{mw_{an}}{c}$, $Q_{in}=\frac{mw_{in}}{c}$, $k_{1}-k_{2}=\Delta k$. $w_{an}$ and $w_{in}$ are the defined resonant frequencies in the anti- and in-phase modes, respectively; $k_{an}$ and $k_{in}$ are the stiffness in the anti- and in-phase modes, respectively; $Q_{an}$ and $Q_{in}$ are quality factors of the ideal anti- and in-phase motions, respectively; and Δk is the mismatch stiffness without the stiffness match.

Thus, Equation (6) can be written as a matrix representation:

(7) $$M\ddot{x}+C\dot{x}+Kx=F\sin wt$$

where $M=\left[\begin{array}{cc} 1 & 0\\ 0 & 1 \end{array}\right]$, $C=\left[\begin{array}{cc} \frac{w_{an}}{Q_{an}} & 0\\ 0 & \frac{w_{in}}{Q_{in}} \end{array}\right]$, $K=\left[\begin{array}{cc} w_{an}^{2} & \frac{\Delta k+k_{e}}{2m}\\ \frac{\Delta k+k_{e}}{2m} & w_{in}^{2} \end{array}\right]$, $F=\left[\begin{array}{c} 0\\ 2a \end{array}\right]$, $x=\left[\begin{array}{c} x_{an}\\ x_{in} \end{array}\right]$.

By using the characteristic equation, the natural frequency can be obtained:

(8) $$\begin{array}{l} w_{1}^{2}=\frac{\left(w_{in}^{2}+w_{an}^{2}\right)-\sqrt{{\left(w_{in}^{2}-w_{an}^{2}\right)}^{2}+{\left(\frac{\Delta k+k_{e}}{m}\right)}^{2}}}{2}\\ w_{2}^{2}=\frac{\left(w_{in}^{2}+w_{an}^{2}\right)+\sqrt{{\left(w_{in}^{2}-w_{an}^{2}\right)}^{2}+{\left(\frac{\Delta k+k_{e}}{m}\right)}^{2}}}{2} \end{array}$$

The modal superposition method is used to solve Equation (7), and the steady-state response is obtained:

(9) $$\begin{array}{c} x(t)=\frac{2\beta_{1}a}{w_{1}^{2}}\cdot \frac{1}{1+{\left(\frac{\left(\Delta k+k_{e}\right)/m}{\sqrt{{\left(w_{in}^{2}-w_{an}^{2}\right)}^{2}+{\left(\frac{\Delta k+k_{e}}{m}\right)}^{2}}+\left(w_{in}^{2}-w_{an}^{2}\right)}\right)}^{2}}\cdot \left[\begin{array}{c} \frac{-\left(\Delta k+k_{e}\right)/m}{\sqrt{{\left(w_{in}^{2}-w_{an}^{2}\right)}^{2}+{\left(\frac{\Delta k+k_{e}}{m}\right)}^{2}}+\left(w_{in}^{2}-w_{an}^{2}\right)}\\ {\left(\frac{\left(\Delta k+k_{e}\right)/m}{\sqrt{{\left(w_{in}^{2}-w_{an}^{2}\right)}^{2}+{\left(\frac{\Delta k+k_{e}}{m}\right)}^{2}}+\left(w_{in}^{2}-w_{an}^{2}\right)}\right)}^{2} \end{array}\right]\;+\;\\ \frac{2\beta_{2}a}{w_{2}^{2}}\cdot \frac{1}{1+{\left(\frac{\left(\Delta k+k_{e}\right)/m}{\sqrt{{\left(w_{in}^{2}-w_{an}^{2}\right)}^{2}+{\left(\frac{\Delta k+k_{e}}{m}\right)}^{2}}-\left(w_{in}^{2}-w_{an}^{2}\right)}\right)}^{2}}\cdot \left[\begin{array}{c} \frac{\left(\Delta k+k_{e}\right)/m}{\sqrt{{\left(w_{in}^{2}-w_{an}^{2}\right)}^{2}+{\left(\frac{\Delta k+k_{e}}{m}\right)}^{2}}-\left(w_{in}^{2}-w_{an}^{2}\right)}\\ {\left(\frac{\left(\Delta k+k_{e}\right)/m}{\sqrt{{\left(w_{in}^{2}-w_{an}^{2}\right)}^{2}+{\left(\frac{\Delta k+k_{e}}{m}\right)}^{2}}-\left(w_{in}^{2}-w_{an}^{2}\right)}\right)}^{2} \end{array}\right] \end{array}$$

where the magnification factor of amplitude $\beta_{i}=\frac{1}{\sqrt{{\left(1-\lambda_{i}^{2}\right)}^{2}+{\left(2\xi_{i}\lambda_{i}\right)}^{2}}}$, the phase angle $\psi_{i}=\arctan\frac{2\xi_{i}\lambda_{i}}{1-\lambda_{i}^{2}}$, the frequency ratio $\lambda_{i}=\frac{w}{w_{i}}$, and the damping ratio $\xi_{i}=\frac{c}{2w_{i}m}$.

When $w=w_{2}$, the displacement difference in the anti-phase mode frequency can be obtained:

(10) $$x_{an}(t)=\frac{2Q_{2}a}{w_{2}^{2}}\cdot \frac{\frac{\left(\Delta k+k_{e}\right)/m}{\sqrt{{\left(w_{in}^{2}-w_{an}^{2}\right)}^{2}+{\left(\frac{\Delta k+k_{e}}{m}\right)}^{2}}-\left(w_{in}^{2}-w_{an}^{2}\right)}}{1+{\left(\frac{\left(\Delta k+k_{e}\right)/m}{\sqrt{{\left(w_{in}^{2}-w_{an}^{2}\right)}^{2}+{\left(\frac{\Delta k+k_{e}}{m}\right)}^{2}}-\left(w_{in}^{2}-w_{an}^{2}\right)}\right)}^{2}}=\frac{2Q_{2}a}{w_{2}^{2}}\cdot \frac{\frac{\Delta k+k_{e}}{\sqrt{{\left(\frac{k_{in}-k_{an}}{2}\right)}^{2}+{\left(\Delta k+k_{e}\right)}^{2}}-\left(\frac{k_{in}-k_{an}}{2}\right)}}{1+{\left(\frac{\Delta k+k_{e}}{\sqrt{{\left(\frac{k_{in}-k_{an}}{2}\right)}^{2}+{\left(\Delta k+k_{e}\right)}^{2}}-\left(\frac{k_{in}-k_{an}}{2}\right)}\right)}^{2}}$$

where $Q_{2}$ is the second-order mode Q-factor.

Considering that $k_{an}-k_{in}>>2\left(\Delta k+k_{e}\right)$ and using the Taylor series expansion:

(11) $$\frac{\Delta k+k_{e}}{\sqrt{{\left(\frac{k_{in}-k_{an}}{2}\right)}^{2}+{\left(\Delta k+k_{e}\right)}^{2}}-\left(\frac{k_{in}-k_{an}}{2}\right)}=\frac{\Delta k+k_{e}}{k_{an}-k_{in}}$$

Substituting Equation (11) into Equation (10):

(12) $$x_{an}(t)=\frac{2Q_{2}a}{w_{2}^{2}}\cdot \frac{\Delta k+k_{e}}{k_{in}-k_{an}}\cos w_{2}t=\frac{Q_{2}a}{2w_{2}^{2}}\cdot \frac{\Delta k+k_{e}}{k^{\prime }}\cos w_{2}t$$

**Figure 2:**
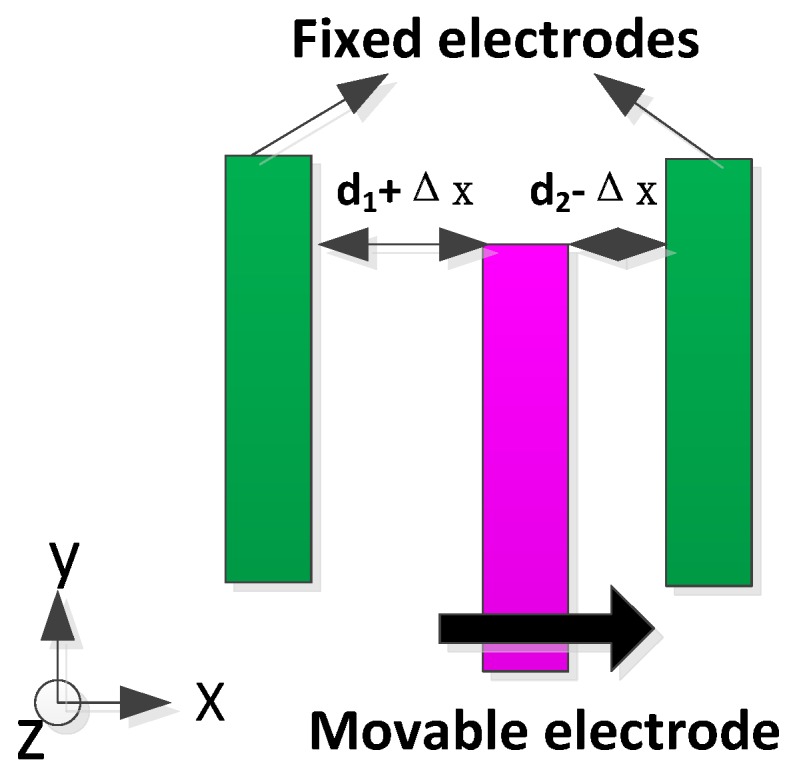
The stiffness match electrodes.

The stiffness mismatch can be reduced using the stiffness match electrodes with the negative electrostatic spring effect [20]. The stiffness match can be implemented by applying a DC voltage in the stiffness match electrodes as shown in Figure 2. The DC voltage of the stiffness match $V_{\Delta k}$ is applied in the fixed electrodes. Assume that the displacement of the mass in the sense direction (along the *x* axis) is Δx. The comb capacitances between the stiffness match electrodes in the left part can be obtained:

(13) $$C_{1}=N\varepsilon \frac{A}{d_{1}+\Delta x},\;C_{2}=N\varepsilon \frac{A}{d_{2}-\Delta x}$$

Thus, the electrostatic force in the left part of the left mass is:

(14) $$F_{e1}=\frac{1}{2}N\varepsilon \varepsilon \left[\frac{1}{{\left(d_{2}-\Delta x\right)}^{2}}-\frac{1}{{\left(d_{1}+\Delta x\right)}^{2}}\right]V_{\Delta k}^{2}$$

Similarly, the electrostatic force in the right part of the left mass is:

(15) $$F_{e2}=\frac{1}{2}N\varepsilon A\left[\frac{1}{{\left(d_{1}-\Delta x\right)}^{2}}-\frac{1}{{\left(d_{2}+\Delta x\right)}^{2}}\right]V_{\Delta k}^{2}$$

where *N* denotes the total number of the stiffness match electrodes.

Therefore, the total electrostatic force is:

(16) $$\begin{array}{cc} F_{e}=F_{e1}+F_{e2}=\frac{1}{2}N\varepsilon A & \left\{\frac{4d_{1}\Delta x}{{\left(d_{1}-\Delta x\right)}^{2}{\left(d_{1}+\Delta x\right)}^{2}}+\frac{4d_{2}\Delta x}{{\left(d_{2}-\Delta x\right)}^{2}{\left(d_{2}+\Delta x\right)}^{2}}\right\}V_{\Delta k}^{2}\\ & \;\approx 2N\varepsilon {\text{AV}}_{\Delta k}^{2}\Delta x\left(\frac{1}{d_{1}^{3}}+\frac{1}{d_{2}^{3}}\right) \end{array}$$

The direction of the electrostatic force is opposite with the direction of the stiffness spring. According to Hooke’s law, the match stiffness $k_{e}$ is a negative stiffness coefficient caused by the electrostatic force:

(17) $$k_{e}=-2N\varepsilon {\text{AV}}_{\Delta k}^{2}\left(\frac{1}{d_{1}^{3}}+\frac{1}{d_{2}^{3}}\right)$$

Therefore, substituting Equation (17) into Equation (12), one obtains:

(18) $$x_{an}(t)=\frac{Q_{2}a}{2w_{2}^{2}}\cdot \frac{\Delta k-2N\varepsilon {\text{AV}}_{\Delta k}^{2}\left(\frac{1}{d_{1}^{3}}+\frac{1}{d_{2}^{3}}\right)}{k^{\prime }}\cos w_{2}t$$

From Equation (18), it is figured out that the anti-phase vibration output can be rejected by reducing the stiffness mismatch through the stiffness match method.

## 3. Design and Fabrication

**Figure 3:**
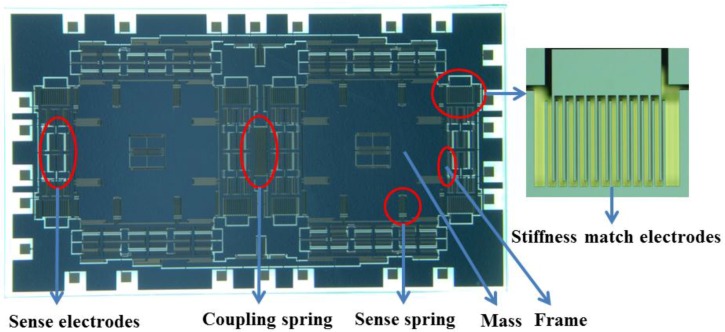
Optical photograph of a dual-mass tuning fork gyroscope.

In order to verify the stiffness match method we proposed, a dual-mass micromachined tuning fork gyroscope is designed and fabricated. The architecture in the sense axis direction (along the horizontal direction) consists of two identical tines and a coupling spring, as shown in Figure 3. Each tine contains a Coriolis mass and a frame, and the Coriolis mass and frame are suspended by the symmetrical springs. These springs except the coupling ones are the same to reduce the stiffness mismatch of the left and right masses caused by the fabrication imperfections. The sense electrodes are variable-area capacitances to ensure the linearity of the capacitance variation with the displacement along the sense direction. The stiffness match electrodes are variable-gap capacitances to reduce the stiffness of one mass for the stiffness match of two masses, as shown in Figure 3.

**Figure 4:**
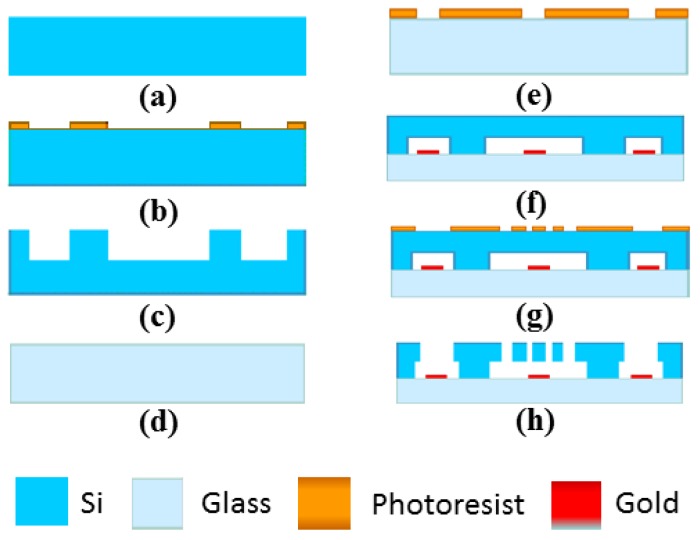
Fabrication process of MEMS tuning fork gyroscope.

The fabrication is implemented using Silicon-on-Glass (SOG) technology. Figure 4 describes the process flow diagram: (a) the silicon wafer was prepared; (b) the photoresist for anchor was patterned; (c) the deep silicon etching was done for forming anchor; (d) the glass wafer was prepared; (e) the photoresist for lift-off was patterned; (f) the electrodes and leads were produced by lift-off and the silicon-glass anodic bonding was performed; (g) the photoresist for structure was patterned; and (h) the deep silicon etching was done for releasing the movable structure. 

**Figure 5:**
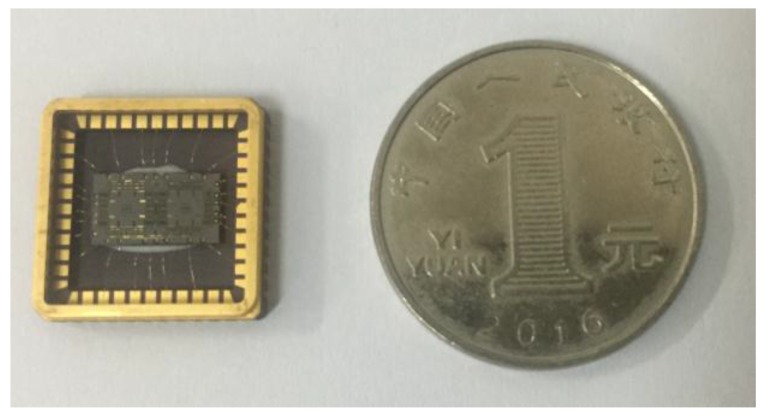
The fabricated MEMS tuning fork gyroscope.

Finally, the fabricated MEMS tuning fork gyroscope is shown in Figure 5.

## 4. Experimental Study and Comparison with Theoretical Model

### 4.1. Experimental Study

**Figure 6:**
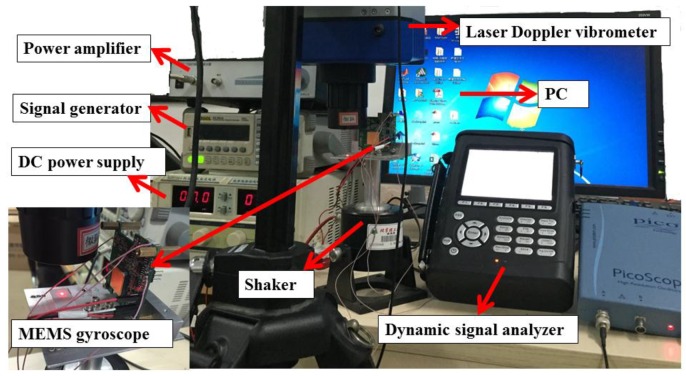
The experimental setup.

In order to further analyze the anti-phase vibration output caused by the linear vibration due to the stiffness mismatch, the experimental study is carried out. The experimental setup is shown in Figure 6. The MEMS gyroscope with the printed circuit board (PCB) is mounted on the vibration shaker, the DC power supply is applied to the circuitry, and the vibration shaker is excited by the excitation signal from the signal generator through the power amplifier. The vibration acceleration signal is tested by the Laser Doppler vibrometer and the corresponding data are acquired by the PC, and the sensing output voltage of the MEMS tuning fork gyroscope is recorded by the dynamic signal analyzer (Crystal Instrument Corporation, Santa Clara, CA, USA, COCO-80).

**Figure 7:**
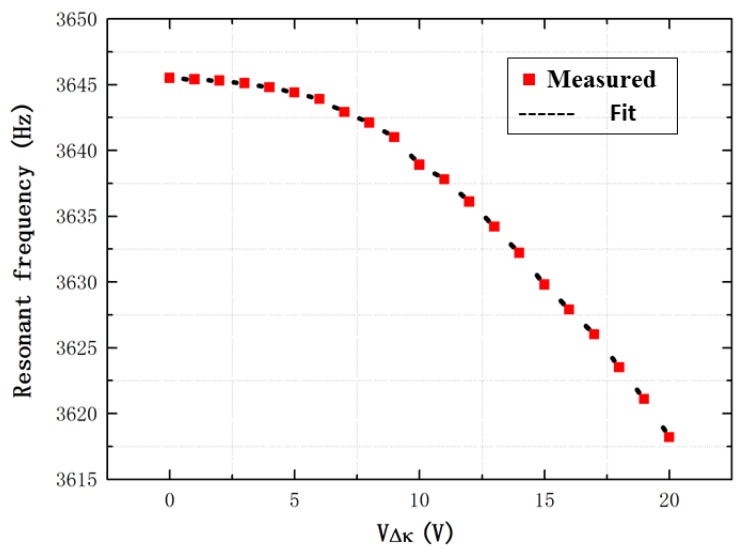
The resonant frequency with the increase of the voltage $V_{\Delta k}$.

**Figure 8:**
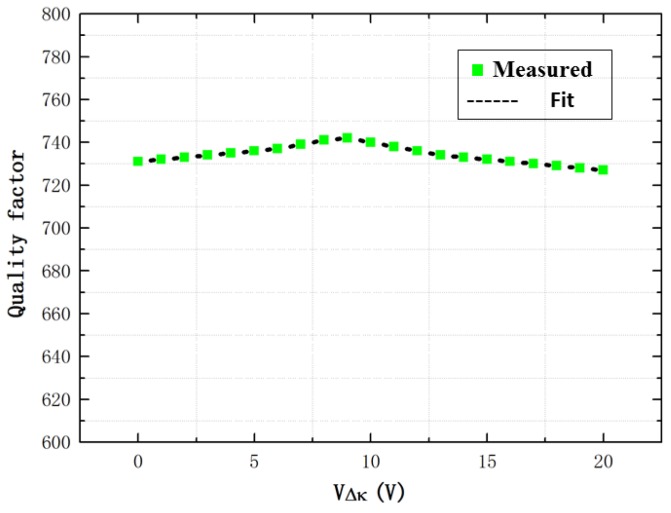
The quality factor with the increase of the voltage $V_{\Delta k}$.

First, the electrical frequency sweep is carried out using the control circuit. Specifically, the movable mass is imposed using a sinusoidal carrier signal of 5 V, and a dc bias voltage of 5 V and an ac voltage of 0.1 V with a frequency sweep range from 3000 to 4000 Hz is applied on the fixed electrodes. To change the degree of the stiffness match, the voltage of the stiffness match $V_{\Delta k}$ imposed on the stiffness match electrodes are from 0 to 20 V. Through the measured frequency response of the MEMS TFG, the anti-phase mode frequency is obtained and the anti-phase Q-factor is acquired by the half-power bandwidth, as shown in Figure 7 and Figure 8, respectively. In Figure 7, the resonant frequency is decreased rapidly with the increase of the voltage $V_{\Delta k}$. The Q-factors are nearly unchanged in Figure 8, which are about 740 under a vacuum.

Figure 7 shows that the anti-phase mode frequency is changed as the voltage of the stiffness match. The purpose is to obtain the resonant frequency under the different voltage of the stiffness match. Because the measured vibration output at the resonant frequency needs to be obtained, it is necessary to first acquire the resonant frequency. Then, the vibration output can be measured at the acquired anti-phase mode frequency.

**Figure 9:**
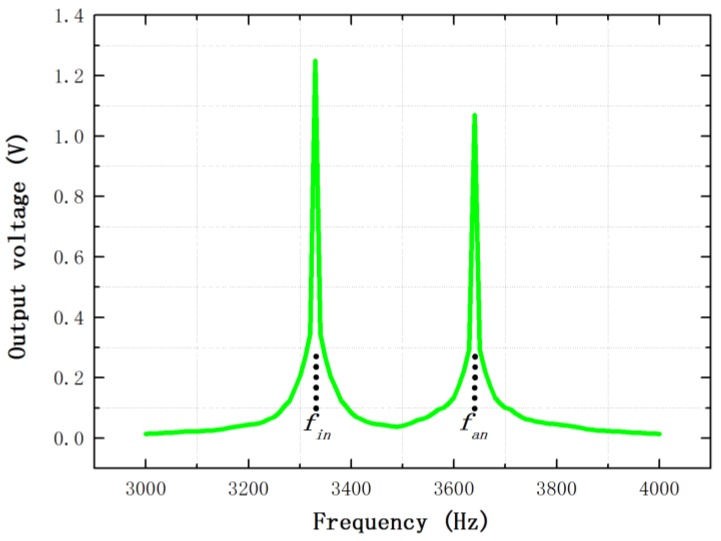
The output voltage of the differential sense capacitance without the stiffness match.

**Figure 10:**
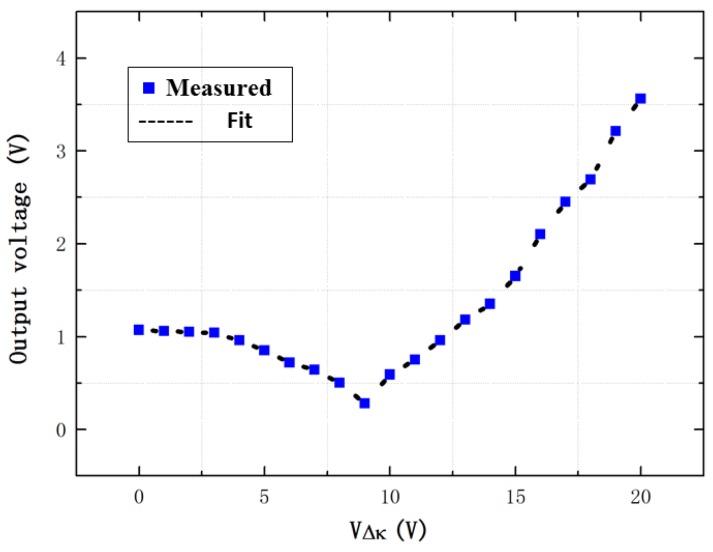
The measured vibration output of the differential sense capacitance in the anti-phase mode frequency.

Then, the mechanical frequency sweep is carried out using a vibration shaker. The printed circuit board is only powered by the DC power supply without the other electrical signals. The excitation acceleration signal with a frequency sweep range from 3000 to 4000 Hz is sinusoidal, and its amplitude is 1 g (9.8 m/s^2^). The output voltage of the differential sense capacitance without the stiffness match is shown in Figure 9. It shows that the vibration output in the in-phase mode and anti-phase mode frequencies are obvious and the in-phase mode frequency $f_{in}$ is 3332.4 Hz while the anti-phase mode frequency $f_{an}$ is 3645.5 Hz. The output voltage of the differential sense capacitance in the anti-phase mode frequency is obtained under the different voltage of the stiffness match $V_{\Delta k}$, as described in Figure 10.

Figure 10 shows that the anti-phase vibration output is firstly decreased and then increased. The anti-phase vibration output is minimized when the voltage $V_{\Delta k}$ is 9 V and the vibration output is 0.28 V, while the anti-phase vibration output is 1.07 V without the stiffness match. Thus, the anti-phase vibration output is reduced by 73.8% compared to the structure without the stiffness match. 

### 4.2. Theoretical Calculation

Due to the practical fabrication defects, the mismatch stiffness Δk is hard to predict. When the anti-phase vibration output is minimized, Δk is same with the negative stiffness coefficient $k_{e}$. Using Equation (17), Δk can be estimated, and the relative stiffness mismatch $\Delta k/k_{2}$, is less than 1.5%. Through the measured in-phase mode frequency $f_{in}$, the anti-phase mode frequency $f_{an}$ and the estimated Δk, the coupling stiffness $k^{\prime }$ and the springs stiffness $k_{2}$ can be solved. The model parameters used in the theoretical model is listed in Table 1. 

According to the measured $f_{an}$ and Q-factor, the estimated Δk and $k^{\prime }$, the theoretical values of the vibration output displacement difference Δx of the differential sense capacitance in the anti-phase mode frequency can be obtained using Equation (18). The capacitance sensitivity $S_{c}$ is 1.427 V/pf and the displacement sensitivity $S_{d}$ is 0.30 pf/um, which are measured by the experimental tests, and expressed as:

(19) $$S_{c}=\frac{V_{0}}{\Delta c},\;S_{d}=\frac{\Delta c}{\Delta x}$$

From Equation (19), the vibration output displacement difference can be expressed as:

(20) $$\Delta x=\frac{V_{0}}{S_{c}\cdot S_{d}}$$

**Figure 11:**
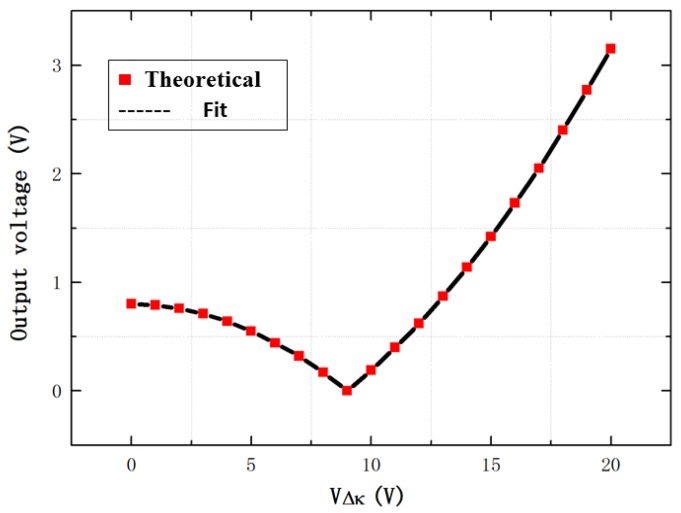
The theoretical vibration output of the differential sense capacitance in the anti-phase mode frequency.

Finally, the theoretical output voltage of the differential sense capacitance in the anti-phase mode frequency is obtained under the different voltage $V_{\Delta k}$, as shown in Figure 11.

### 4.3. Experimental and Theoretical Comparisons

**Figure 12:**
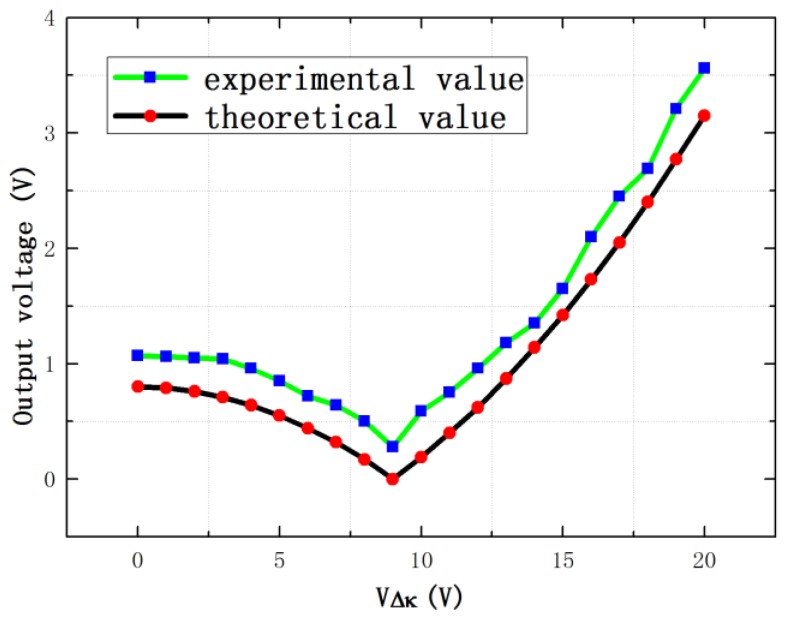
Comparisons with experimental and theoretical values of the vibration output.

Comparisons of the experimental and theoretical values are displayed in Figure 12. As can be seen in Figure 12, there is an error between the experimental and theoretical values and the theoretical value is a little smaller than the experimental value. The variation tendency is almost the same, which experimentally verifies the proposed stiffness match method. According to the previous theoretical Equation (18) (described in Section 2), the vibration output is zero when the stiffness of the left mass and the right mass is matched, while the measured minimum value is 0.28 V. The possible reasons are that the mass and damping of the left and right tines are also mismatch and other possible causes, which will induce the anti-phase vibration output. 

## 5. Discussion

According to Equation (18), it is concluded that the anti-phase vibration output of the MEMS tuning fork gyroscope is proportional to the stiffness mismatch and inversely proportional to the coupling stiffness. Based on the theoretical analysis, it can be seen that there are two ways to effectively eliminate the anti-phase vibration output without sacrificing the scale factor. One way is to design a structure to increase the coupling stiffness by using the anchored coupling mechanism according to the previous study [16], which takes advantage of the structural approach to reduce the vibration output. Another way is to design stiffness match electrodes with the corresponding circuit to reduce the stiffness mismatch, which makes use of the control circuit approach based on applying a DC voltage on the stiffness match electrodes to eliminate the vibration output with the negative electrostatic spring effect.

However, the anti-phase vibration output is still not completely eliminated. The possible reasons are that there are also mismatch between the mass and damping of the left and right tines and the other possible causes such as the asymmetric electrostatic forces and the capacitance nonlinearity, which will induce the anti-phase vibration output. Because other complicated factors can affect the anti-phase vibration output, how to better reduce the anti-phase vibration output needs to be further studied in the future.

## 6. Conclusions

This paper proposes a stiffness match method to reduce the vibration sensitivity of micromachined tuning fork gyroscopes. Making use of the coordinate transformation method, a theoretical model is set up to investigate the anti-phase vibration output caused by the stiffness mismatch due to the fabrication imperfections. The analytical results reveal that the stiffness mismatch is proportional to the output induced by the linear vibration from the sense direction in the anti-phase mode frequency. In order to verify the proposed stiffness match method, the TFG including the stiffness match electrodes are designed and implemented using micromachining technology and the experimental study is carried out. The experimental tests demonstrate that the linear vibration output can be reduced by 73.8% through the stiffness match method with stiffness match electrodes compared to the structure without the stiffness match. Therefore, the proposed stiffness match method is experimentally validated to be applicable to the vibration sensitivity reduction in the tuning fork gyroscopes without sacrificing the sensitivity.

## Figures and Tables

**Table 1:** Parameters used in the theoretical model.

Parameters	Value	Parameters	Value
Sense-mode mass	1.3951 × 10^−6^ kg	Structural thickness	80 µm
Mismatch stiffness	8.1 N/m	Sense-mode Q	740
Springs stiffness k_2_	607 N/m	Common acceleration	9.8 m/s^2^
Stiffness mismatch	1.33%	Coupling stiffness	60 N/m
